# EPH/ephrin profile and EPHB2 expression predicts patient survival in breast cancer

**DOI:** 10.18632/oncotarget.7246

**Published:** 2016-02-08

**Authors:** Anna-Maria Husa, Željana Magić, Malin Larsson, Tommy Fornander, Gizeh Pérez-Tenorio

**Affiliations:** ^1^ Department of Clinical and Experimental Medicine, Division of Oncology, Linköping University, Linköping, Sweden; ^2^ Current address: CCRI, Children's Cancer Research Institute, St. Anna Kinderkrebsforschung e.V., Vienna, Austria; ^3^ Bioinformatics Infrastructure for Life Sciences (BILS) and Department of Physics, Chemistry and Biology, Linköping University, Linköping, Sweden; ^4^ Department of Oncology, Karolinska University Hospital and Karolinska Institute, Stockholm, Sweden

**Keywords:** EPHB2, EPH family, TaqMan array, gene expression, protein expression

## Abstract

The EPH and ephrins function as both receptor and ligands and the output on their complex signaling is currently investigated in cancer. Previous work shows that some EPH family members have clinical value in breast cancer, suggesting that this family could be a source of novel clinical targets. Here we quantified the mRNA expression levels of EPH receptors and their ligands, ephrins, in 65 node positive breast cancer samples by RT-PCR with TaqMan^®^ Micro Fluidics Cards Microarray. Upon hierarchical clustering of the mRNA expression levels, we identified a subgroup of patients with high expression, and poor clinical outcome. *EPHA2, EPHA4, EFNB1, EFNB2, EPHB2* and *EPHB6* were significantly correlated with the cluster groups and particularly *EPHB2* was an independent prognostic factor in multivariate analysis and in four public databases. The EPHB2 protein expression was also analyzed by immunohistochemistry in paraffin embedded material (cohort 2). EPHB2 was detected in the membrane and cytoplasmic cell compartments and there was an inverse correlation between membranous and cytoplasmic EPHB2. Membranous EPHB2 predicted longer breast cancer survival in both univariate and multivariate analysis while cytoplasmic EPHB2 indicated shorter breast cancer survival in univariate analysis. Concluding: the *EPH/EFN* cluster analysis revealed that high *EPH/EFN* mRNA expression is an independent prognostic factor for poor survival. Especially *EPHB2* predicted poor breast cancer survival in several materials and EPHB2 protein expression has also prognostic value depending on cell localization.

## INTRODUCTION

Breast cancer prognosis and treatment mostly relies on a few markers such as the estrogen receptor (ER), progesterone receptor (PgR), the human epidermal growth factor receptor 2 (HER2/neu) and tumor stage. Positive ER and PgR expression helps identifying patients more likely to benefit from endocrine treatment while HER2 over expression and amplification predicts response to trastuzumab and lapatinib [1, 2]. Despite advances in breast cancer prognosis and treatment [2, 3] some patients have a short disease-free survival period demonstrating the need of better clinical markers.

Therefore we proposed to screen the EPH receptor family and its ligands. The EPH receptors belong to the largest family of tyrosine kinase receptors with implications in cancer [4-6]. The name EPH derived from an Erythropoietin-Producing Hepatocellular carcinoma cell line used to clone the receptor for the first time [7, 8]. EPH receptors, together with membrane-bound ligands (ephrins) play a crucial role not only in mammary gland development but also in carcinogenesis [9]. EPH and ephrins influence cell adhesion, cell migration, intercellular junction formation, cell shape, cell motility, cell guidance and pattern formation [10, 11].

The EPH family is composed of subclasses A and B, based on sequence homology, and structural features. EPHA receptors are attached to the plasma membrane by a glycosylphosphatidylinositol tail and preferentially bind ephrin-A ligands. EPHB receptors have a single trans-membrane domain and a short cytoplasmic tail and usually recognize transmembrane ephrin-B ligands [12]. Upon cell-to-cell contact, EPH receptors and ephrins interact and transduce signals in a bidirectional manner. Bidirectional signals are defined as “forward signals” when deriving from EPH receptors present in epithelial cells and “reverse signals” when transmitted by the ephrin ligands expressed by, for example, endothelial cells. The EPH-ephrin interaction is usually restricted to members of the same class but hetero-dimerization between EPHA and EPHB members and ephrins takes place [13].

EPH receptors can also “cross talk” with other signaling molecules [14] and receptor tyrosine kinases (RTK) [15]. Therefore, it is believed that EPH-ephrin interactions are complex and promiscuous affecting both the normal and malignant epithelium [9].

Among the family members, EPHA2 and EPHB4 are the most studied in breast cancer and additionally EPHA4, EPHA7 and EPHB6 emerged as promising clinical candidates in an expression profile of the individual EPH and ephrin family members [16]. Here, we explored whether a cluster analysis of the EPH/ephrin gene expression levels would reveal patient subgroups with different clinical outcome. For this purpose the *EPH/EFN* gene expression was quantified using TaqMan^®^ Array Micro Fluidics Cards containing 21 EPH/ephrin family members and then proceeded to group the patients based on their gene expression levels. This approach, which differs from the one used in a previous study [16], allowed us identifying a subgroup of patients with higher expression levels of the *EPH*/*EFN* genes and more frequent relapse of the disease compared with the rest of the patients. Also, in addition to the previous report, we found that *EFNB1*, *EFNB2* and *EPHB2* were interesting candidates due to the strong correlation between these genes and the cluster groups. *EPHB2* was identified as an independent prognostic factor in multivariate analysis and therefore we also investigated the expression of EPHB2 at the protein level. EPHB2 was found in the cell membrane and the cytoplasm of the tumor cells. However, membranous EPHB2 and cytoplasmic EPHB2 were inversely correlated indicating different patient prognosis. Positive membranous EPHB2 was coupled to better prognosis while cytoplasmic EPHB2 was associated with shorter disease-free survival. This finding suggests that the EPHB2 cellular localization introduces another level of complexity.

In conclusion, we confirmed the clinical value of EPHA2, EPHB4 and EPHB6. We also suggest that EFNB1 and EFNB2 could be additional interesting candidates and revealed the clinical value of EPHB2 as a potential prognostic marker in breast cancer.

## RESULTS

### Expression of the *EPH/EFN* gene family (cohort 1)

**Figure 1:**
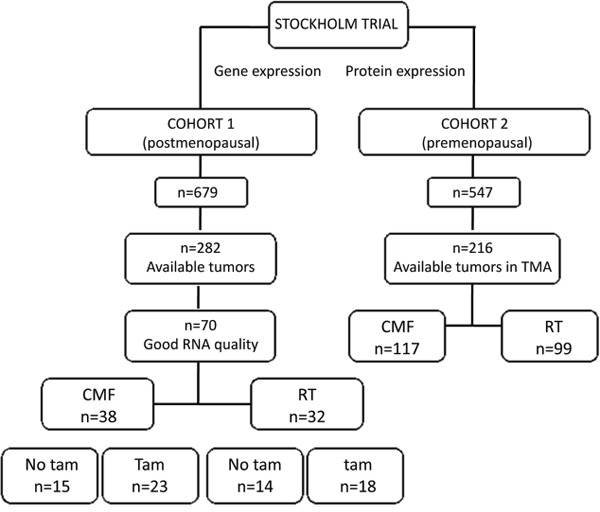
Patient distribution in the two cohorts included in this study The treatments consisted in cyclophosphamide, methotrexate and 5-fluorouracil (CMF), radiation therapy (RT) or tamoxifen (Tam).

**Figure 2:**
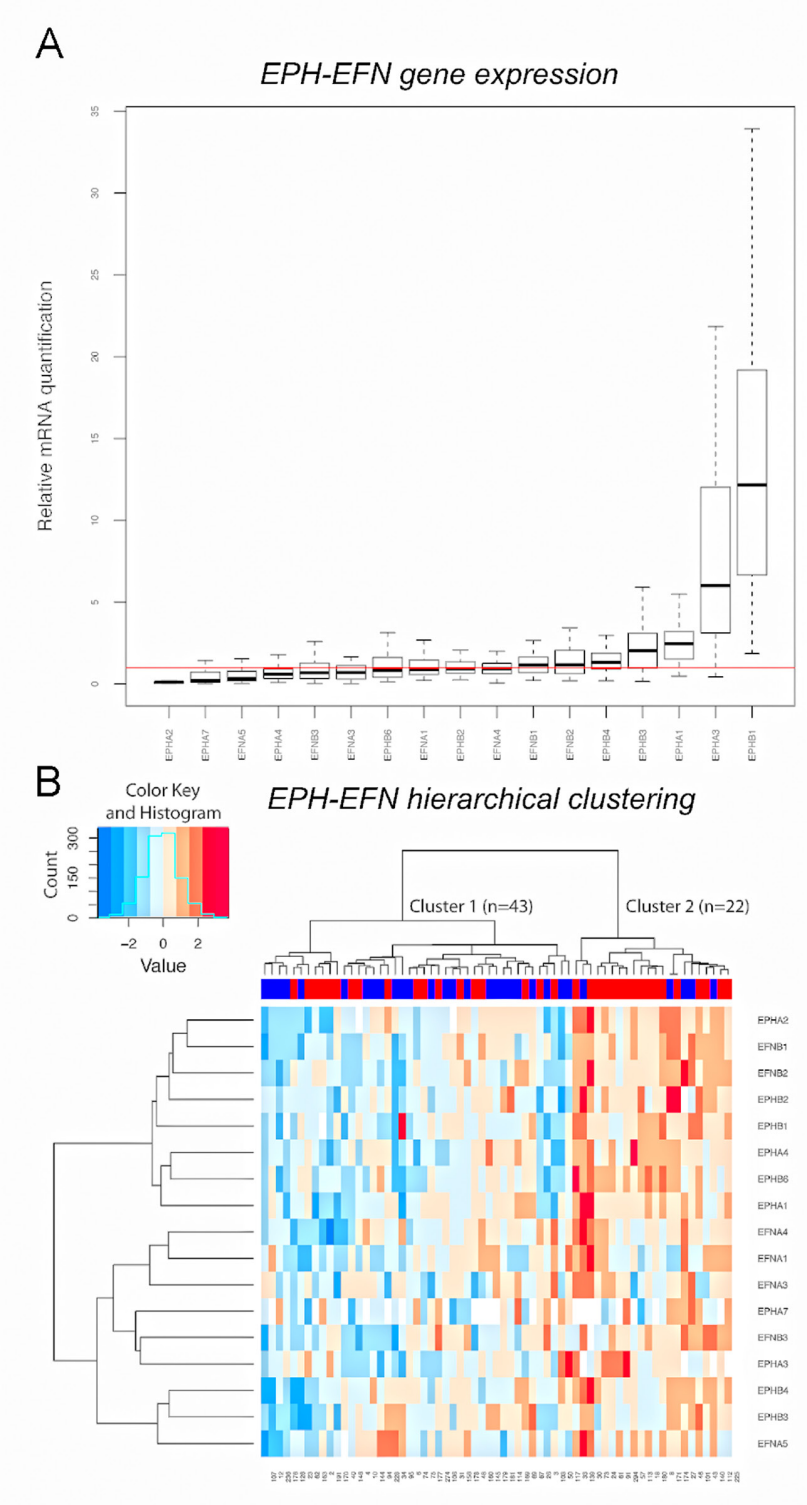
*EPH/EFN* mRNA expression in breast cancer patients with lymph nodal infiltration **A.** The boxes show the distributions of *EPH/EFN* relative mRNA expression levels across the breast cancer samples, normalized to the endogenous control *HPRT1* and relative to the expression levels in the cell line pool. The red line shows y=1 which corresponds to the relative expression levels of the EPH/EFN genes in the cell line pool. The box plot shows expression levels for those EPH family members that were expressed at detectable levels in clinical samples. Hierarchical clustering showing that the patients were clustered into cluster 1 (n=43) with low to medium mRNA levels of the EPH family members and cluster 2 (n=22) with high mRNA expression **B.** Numbers below the heat map represent anonymous patient identification. The red inserts in the upper blue bar indicate total recurrences. The color key for the mRNA expression is blue for low to medium expression and red for high expression. Both graphs were built in R.

Gene expression levels were quantified in the first patient cohort (Fig. 1). All analyzed genes expressed mRNA at detectable levels in the cell pool used as reference sample. More than 90% of the tumors expressed mRNA for *EFNA1, EFNA2, EFNA3, EFNA4, EFNA5, EFNB1, EFNB2, EFNB3, EPHA1, EPHA2, EPHA3, EPHA4, EPHA7, EPHB1, EPHB2, EPHB3, EPHB4* and *EPHB6*. However, mRNA levels for *EPHA5, EPHA6* and *EPHA8* were detected in <40% of the tumors, and although mRNA for *EFNA2* was present in most tumors it was poorly expressed with high variance. Relative mRNA expression levels of the analyzed genes, except for *EPHA5, EPHA6, EPHA8 and EFNA2,* are shown in Fig. 2A. *EPHB1* showed the highest relative mRNA expression in the breast cancer samples and *EPHA2* the lowest.

### Cluster and statistical analyses (cohort 1)

Unsupervised hierarchical clustering was used to group the patients in cohort 1 according to their expression levels of the *EPH/EFN* gene family. In order to have a clinically homogenous cohort, only the 65 patients with lymph node infiltration were included. The hierarchical clustering divided the patients in two main clusters. The patients in the smaller cluster (n=22), generally expressed the *EPHA2, EFNB1, EFNB2, EPHB2, EPHB1, EPHA4, EPHB6, EPHA1, EFNA4, EFNA1, EFNA3, EPHA7, EFNB3, EPHB4, EPHB3 and EFNA5* genes at higher levels in comparison with the patients in the larger cluster 1 (n=43) (Fig. 2B).

A categorical variable was assigned to each patient describing whether it belonged to the “high expression” cluster or cluster 2 (34% of the patients) or to the “low expression” cluster or cluster 1 (66% of the patients). Spearman Rank Correlation was then used to test the correlation between cluster groups and the expression levels of individual EPH genes. It was noted that the strongest correlation with the cluster groups was observed for *EPHA2, EFNB1, EFNB2, EPHB2, EPHA4* and *EPHB6* (P<0.000001) indicating that these genes are the most representative members of this cluster. However, no other known clinical variable was associated with the “cluster groups” categorical variable (Table 1). Among the EPH members, *EPHB2* mRNA expression was positively associated with HER2 protein expression.

**Table 1:** Correlation between the cluster groups, *EPHB2* (median) and other clinical variables in Cohort 1

	Cluster 1 n (%)	Cluster 2 n (%)	P value	*EPHB2*- n (%)	*EPHB2*+ n (%)	P value
**Tumor Size**						
<20 mm	16 (62)	10 (38)		11 (42)	15 (58)	
≥20 mm	27 (69)	12 (31)	0.53	19 (50)	19 (50)	0.55
**ERα**^a^						
−	10 (67)	5 (33)		7 (47)	8 (53)	
+	33 (66)	17 (34)	0.96	23 (47)	26 (53)	0.99
**HER2 protein**^b^						
-	34 (68)	16 (32)		27 (55)	22 (45)	
+	8 (57)	6 (43)	0.46	2 (14)	12 (86)	**0.006**
**ERBB2 gene**^c^						
Non amplified	36 (68)	17 (32)		28 (54)	24 (46)	
Amplified	5 (56)	4 (44)	0.47	1 (11)	8 (89)	0.02
**Treatment**^d^						
-	7 (54)	6 (46)		6 (46)	7 (54)	
+	36 (69)	16 (31)	0.30	24 (47)	27 (53)	0.95
**SPhase (%)**^e^						
< 10	24 (73)	9 (27)		19 (58)	14 (42)	
≥ 10	17 (61)	11 (39)	0.33	9 (32)	19 (68)	0.05
**pAKT (Ser473)**^f^						
-	18 (55)	15 (45)		14 (42)	19 (58)	
+	25 (78)	7 (22)	0.045	16 (52)	15 (48)	0.47
***EPHB2***						
-	28 (93)	2 (7)				
+	15 (44)	19 (56)	**0.0001**			

a Estrogen receptor alpha (ERα) measured by isoelectric focusing with cutoff value of 0.05 fmol/μg DNA,

b Determined by flow cytometry [41],

c [40],

d Treatment − (radiotherapy) Treatment + (cytostatics alone or together with tamoxifen or radiotherapy together with tamoxifen.

e S phase Fraction,

f [41]. Significant P≤0.01 in bold adjusted for multiple comparisons.

#### Survival analysis

**Figure 3:**
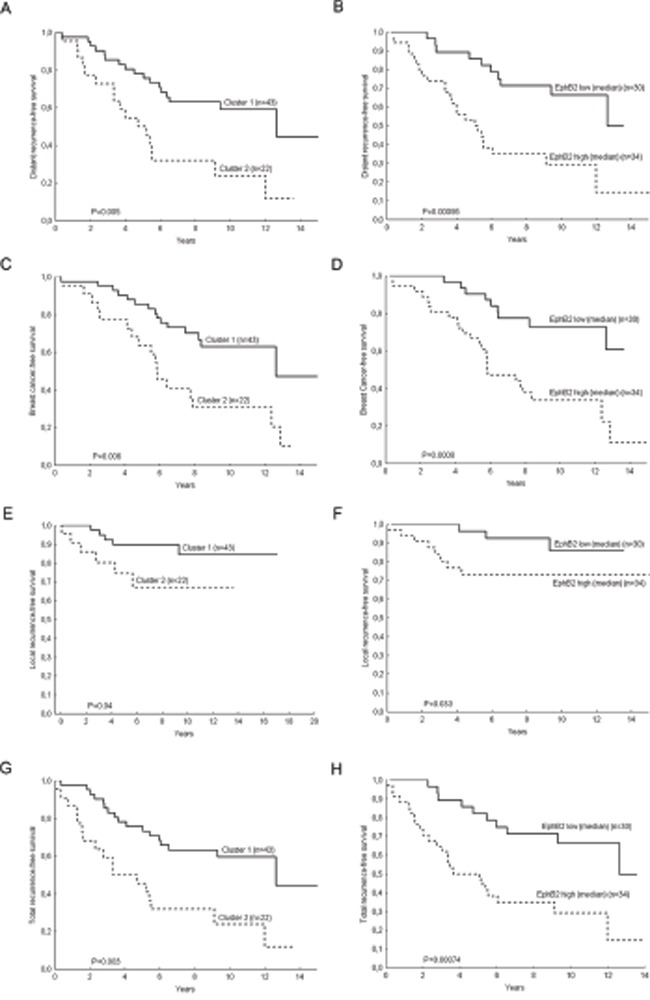
Kaplan-Meier Plots for breast cancer patients with lymph nodal infiltration Patients who belong to the cluster group 2 have shorter distant recurrence-free survival (H.R. (95%CI)=2.7 (1.39-5.39), P=0.004) **A.** shorter breast cancer survival (H.R. (95%CI)=2.79 (1.39-5.63), P=0.004) **C.** shorter local recurrence-free survival (H.R. (95%CI)=3.06 (0.93-10.09), P=0.07) **E.** and total recurrence-free survival (H.R. (95%CI)=2.85 (1.45-5.60), P=0.002) **G.** Likewise the patients with high *EPHB2* mRNA expressing tumors have shorter distant recurrence-free survival (H.R. (95%CI)=3.31 (1.57-7.01), P=0.002) **B.** breast cancer-free survival (H.R. (95%CI)= 3.72 (1.66-8.30), P=0.001) **D.** local recurrence-free survival (H.R. (95%CI)=3.23 (0.85-12.3), P=0.09) **F.** and total recurrence-free survival (H.R. (95%CI)=3.3 (1.56-6.97), P=0.002) **H.** P values in the plots correspond to the Gehan's Wilcoxon test and Hazard Ratios to the Cox regression analysis.

Univariate Cox proportional Hazard Regression and the Gehan's Wilcoxon test (included in the Kaplan-Meier plots) were used to assess whether there were differences in recurrence-free survival time for the patients in the cluster 2 compared with the patients in the cluster 1. Four end-points were analyzed: distant recurrence-free survival, breast cancer-survival, local recurrence-free survival and total recurrence-free survival (time from surgery to development of local or distant recurrences) (Fig. 3A, 3C, 3E, 3G).

Furthermore, a multivariate Cox analysis (Table 2) showed the independent prognostic value of the cluster groups and *EPHB2* with the covariates treatment, tumor size, HER2 protein expression and ERα. *EPHB6*, which also had a high impact on the cluster separation, had independent prognostic value in univariate analysis. However in multivariate analysis with the covariates *EPHB2* and the cluster groups, only *EPHB2* remained significant (Table 3 and Fig. 3B, 3D, 3F, 3H).

**Table 2:** Multivariate Cox proportional hazard regression model for cohort 1

Variables	Breast Cancer death		Distant Recurrences		Total Recurrences	
	H.R. (95% CI)^a^	P-value	H.R. (95% CI)	P-value	H.R. (95% CI)	P-value
Treatment^b^						
−	1.00		1.00		1.00	
+	0.81 (0.31-2.13)	0.679	0.73 (0.29-1.83)	0.51	0.81 (0.33-2.03)	0.658
Tumor size (mm)						
< 20	1.00		1.00		1.00	
≥ 20	0.79 (0.39-1.63)	0.520	0.75 (0.37-1.51)	0.41	0.75 (0.37-1.53)	0.433
HER2 protein^c^						
−	1.00		1.00		1.00	
+	1.20 (0.49-2.91)	0.693	1.19 (0.49-2.88)	0.697	1.20 (0.49-2.95)	0.696
ERα^d^						
−	1.00		1.00		1.00	
+	0.79 (0.32-1.94)	0.600	0.84 (0.36-1.94)	0.678	0.80 (0.33-1.92)	0.611
Cluster groups						
1	1.00		1.00		1.00	
2	2.76 (1.33-5.73)	**0.007**	2.63 (1.29-5.36)	**0.008**	2.80 (1.37-5.72)	**0.005**
	**H.R. (95% CI)**	**P-value**	**H.R. (95% CI)**	**P-value**	**H.R. (95% CI)**	**P-value**
Treatment						
-	1.00		1.00		1.00	
+	1.03 (0.41-2.62)	0.938	0.91 (0.38-2.20)	0.840	0.97 (0.40-2.33)	0.946
Tumor size (mm)						
< 20	1.00		1.00		1.00	
≥ 20	1.08 (0.53-2.23)	0.822	0.95 (0.48-1.91)	0.895	0.96 (0.47-1.95)	0.910
HER2 protein						
-	1.00		1.00		1.00	
+	0.76 (0.31-1.85)	0.544	0.86 (0.35-2.08)	0.731	0.87 (0.35-2.14)	0.759
ERα						
-	1.00		1.00		1.00	
+	1.04 (0.42-2.52)	0.939	1.09 (0.47-2.51)	0.847	1.04 (0.44-2.48)	0.923
*EPHB2*						
Low	1.00		1.00		1.00	
High	3.84 (1.69-8.73)	**0.001**	3.11 (1.44-6.72)	**0.004**	3.10 (1.44-6.70)	**0.004**
Systemic treatment						
-	1.00		1.00		1.00	
+	1.00 (0.36-2.74)	0.99	0.92 (0.34-2.48)	0.87	0.95 (0.36-2.53)	0.92
Tumor size (mm)						
< 20	1.00		1.00		1.00	
≥ 20	1.03 (0.49-2.15)	0.94	0.90 (0.44-1.84)	0.78	0.91 (0.44-1.87)	0.80
HER2 protein						
-	1.00		1.00		1.00	
+	1.22 (0.51-2.93)	0.65	1.39 (0.58-3.30)	0.46	1.36 (0.57-3.25)	0.49
ERα						
-	1.00		1.00		1.00	
+	1.39 (0.55-3.51)	0.49	1.49 (0.60-3.69)	0.39	1.40 (0.56-3.48)	0.47
*EPHB6*						
Low	1.00		1.00		1.00	
High	3.05 (1.38-6.72)	**0.006**	2.98 (1.34-6.64)	**0.007**	2.80 (1.28-6.13)	**0.01**

a Hazard Ratio (H.R.) and confident intervals calculated with the Cox model,

b Treatment − (radiotherapy) Treatment + (cytostatics alone or together with tamoxifen or radiotherapy together with tamoxifen),

c HER2 protein measured by flow cytometry,

d estrogen receptor alpha (ERα) measured by isoelectric focusing, cutoff value of 0.05 fmol/μg DNA. Significant P≤0.01 in bold adjusted for multiple comparisons.

**Table 3:** Multivariate Cox proportional hazard regression model with Cluster groups, *EPHB2* and *EPHB6* as additional covariates

Variables	Breast cancer death		Distant recurrences		Total recurrences	
	H.R. (95% CI)^a^	H.R. (95% CI)	H.R. (95% CI)	P-value	H.R. (95% CI)	P-value
Systemic treatment^b^						
-	1.00		1.00		1.00	
+	1.34 (0.47-3.79)	0.58	1.22 (0.43-3.44)	0.71	1.29 (0.47-3.58)	0.62
Tumor size (mm)						
< 20	1.00		1.00		1.00	
≥ 20	1.13 (0.53-2.41)	0.75	1.00 (0.49-2.08)	0.99	0.96 (0.46-2.00)	0.92
HER2 protein^c^						
-	1.00		1.00		1.00	
+	0.65 (0.25-1.74)	0.39	0.77 (0.29-2.03)	0.59	0.73 (0.27-2.00)	0.54
ERα^d^						
-	1.00		1.00		1.00	
+	0.87 (0.31-2.39)	0.78	0.98 (0.37-2.66)	0.98	0.83 (0.30-2.34)	0.73
Cluster groups						
1	1.00		1.00		1.00	
2	1.42 (0.56-3.61)	0.47	1.49 (0.57-3.87)	0.41	1.73 (0.68-4.45)	0.25
*EPHB2*^e^						
Low	1.00		1.00			
High	3.21(1.18-8.72)	**0.02**	2.60 (1.02-6.61)	**0.04**	2.62 (1.03-6.63)	**0.04**
*EPHB6*^f^						
Low	1.00		1.00			
High	1.90 (0.74-4.89)	0.18	1.76 (0.64-4.87)	0.28	1.54 (0.58-4.09)	0.39

a Hazard Ratio (H.R.) and confident intervals calculated with the Cox proportional hazard regression model,

b systemic treatment − (radiotherapy), systemic treatment + (cytostatics, tamoxifen or cytostatics + tamoxifen),

c HER2 protein measured by flow cytometry,

d estrogen receptor alpha (ERα) cutoff value of 0.05 fmol/μg/DNA,

e EPHB2 Low (≤median) EPHB2 High (> median),

f EPHB6 Low (quartiles 1-3) EPHB6 High (quartile 4).

**Figure 4:**
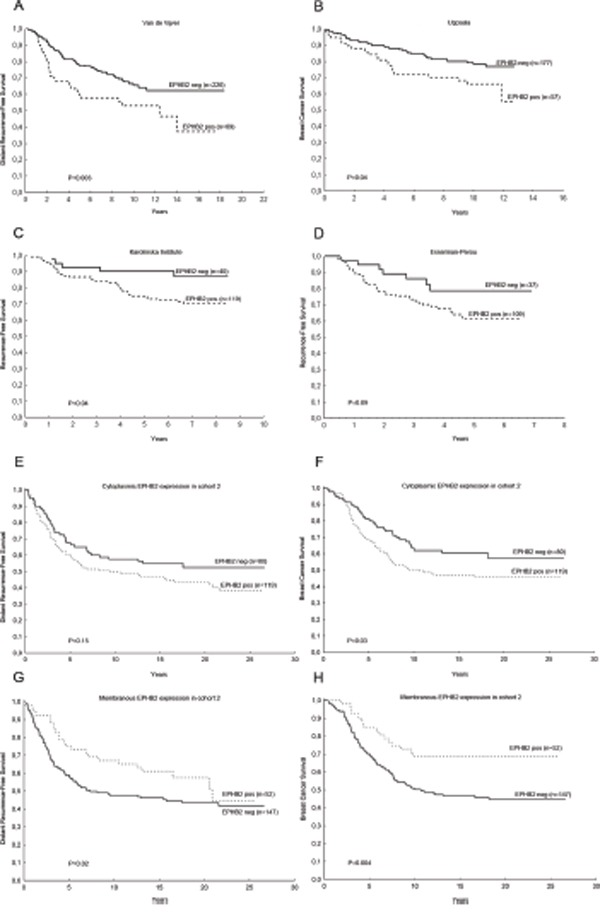
The clinical significance of *EPHB2* mRNA expression was validated in public databases and at the protein level in a second patient cohort. High *EPHB2* mRNA expression predicted shorter distant recurrence-free survival (H.R. (95%CI)=1.78(1.18-2.71), P=0.006) **A.** shorter breast cancer survival **B.** (H.R. (95%CI)=1.76 (1.01-3.05), P=0.04), trend to shorter recurrence-free survival **C.** (H.R. (95%CI)=2.59 (1.02-6.62), P=0.05) and (H.R. (95%CI)=1.91 (0.85-4.29), P=0.12 **D.** In the second cohort, high EPHB2 cytoplasmic expression borderline predicted shorter distant recurrence-free survival, H.R. (95%CI)=1.37 (0.92-2.04), P=0.12 **E.** and poor breast cancer survival, H.R. (95%CI)=1.52 (0.10-2.33), P=0.05 **F.** While, high membranous EPHB2 indicated longer distant recurrence-free survival, (H.R. (95%CI)=0.66 (0.41-1.05), P=0.08 **G.** and longer breast cancer survival, (H.R. (95%CI)=0.47 (0.28-0.80), P=0.006 **H.** Hazard Ratios (H.R.) were calculated with the Cox regression analysis while P values included in the plots correspond to the Gehan's Wilcoxon test.

We next explored the potential clinical value of EPHB2 in four public datasets (Fig. 4A-4D) finding that EPHB2 has prognostic value in other patient cohorts and even for patients without lymphnodal infiltration. Therefore we continued exploring the role of EPHB2 at the protein level in a larger patient material (cohort 2, Fig. 1).

### EPHB2 protein expression (cohort 2)

**Figure 5:**
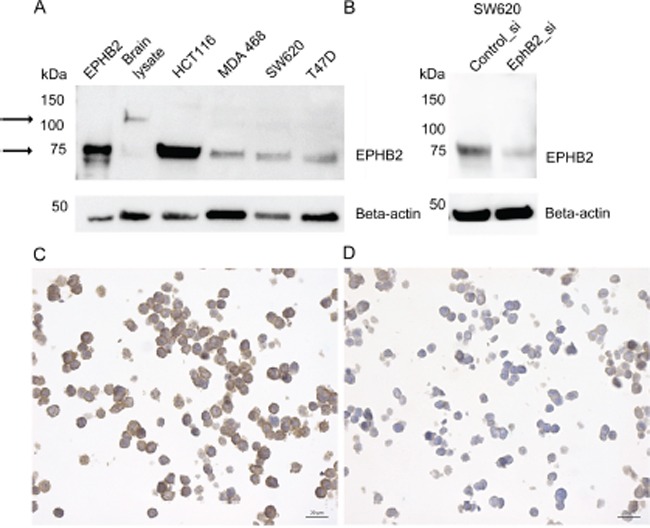
EPHB2 Antibody validation by immunoblot and immunocytochemistry The left arrows indicate bands at ≈ 100 kDa (brain lysate) and 75kDa (all other cell lines and the positive control). The EPHB2 positive control consisted in HEK293 expressing the recombinant human EPHB2/Fc extracellular domain with estimated Mw between 50-90 kDa. Other tested lysates were extracted from mouse brain, human colorectal cancer cells HCT116 and SW620 and breast cancer cells MDA-MB-468 and T47D **A.** The SW620, a colon cell lined derived from a metastatic lymph node, was selected to knockdown EPHB2 expression with siRNA **B.** or treated with the AllStars control siRNA. Beta actin (1:1000) was used as loading control. Paraffin embedded HCT116 cells treated with control siRNA **C.** or EPHB2 siRNAs **D.** were stained with the rabbit anti-EPHB2 (dilution 1:300).

EPHB2 protein expression was determined by immunohistochemistry with a polyclonal rabbit anti-EPHB2 antibody raised against the recombinant EPHB2. A commercial cell lysate from HEK293 expressing the extracellular human EPHB2-Fc domain was used as positive control for the immunoblot. Also cell lysates from mouse brain and human colorectal cancer cells (HCT116, SW620) with reported EPHB2 expression, were used. Additionally we detected EPHB2 in MDA-MB-231, MDA-MB-468 and T47D breast cancer cells. The immunoblot (Fig. 5A) shows that the antibody recognizes a single protein band in all the samples including the HEK293 positive control. In the brain lysate the detected band matches the predicted molecular weight slightly above 100 kDa. In the cancer cells, however, a 75kDa band was visualized. This band was also EPHB2 as proved by LC-MS/MS analysis (see Supplementary Methods, Supplementary Table S3 and Supplementary Fig. S1). To further validate the antibody; in addition of using a blocking peptide (result not shown), the SW620 cells were selected to knock down the EPHB2 expression with siRNAs. Fig. 5B shows that the EPHB2 (75kDa) is detected by the rabbit polyclonal antibody in the control siRNA-treated cells but hardly in the EPHB2 siRNA-treated cells due to knockdown of the EPHB2 protein in these cells.

Also to prove that the anti-EPHB2 was specific and suitable for studies in paraffin embedded material, we used paraffin-embedded HCT116 cells pre-treated with control or EPHB2 siRNAs. A strong membranous staining was observed in the control cells (Fig. 5C) compared to a negative/weak EPHB2 signal in the knockdown cells (Fig. 5D).

**Figure 6:**
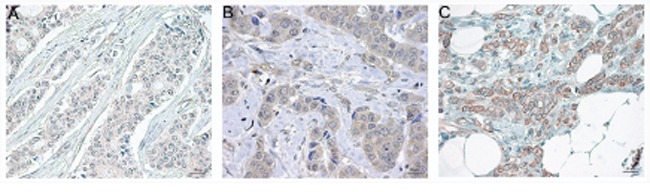
Representative EPHB2 immunostaining in paraffin embedded breast cancer tissues at a final magnification of 630 X Negative staining **A.** positive cytoplasmic staining **B.** and positive membranous staining **C.** The Image analysis was performed with the software Aperio ImageScope v.12.2 and the panels were assembled in Adobe Photoshop CS5 extended v.12.

Regarding the clinical material, some tumors were negative for EPHB2 (Fig. 6A) while other tumors showed cytoplasmic staining (Fig. 6B) or membrane staining (Fig. 6C). For the cytoplasmic staining, 60% of the tumors were classified as positive (scored as C>0), and 26% presented strong membranous staining (scored as M=2). Only 7% of the tumors presented nuclear staining (result not shown).

Cytoplasmic EPHB2 was positively correlated with Nottingham histological grade (NHG), P=0.0008 and with HER2 protein expression, P=0.01 and borderline negatively correlated to membranous EPHB2 expression, P=0.03. Membranous EPHB2 expression did not correlate with any other clinical variable except for cytoplasmic EPHB2 (Table 4).

**Table 4:** EPHB2 protein expression in relation to known clinical variables in Cohort 2

	EPHB2_C-	EPHB2_C+^a^	P value	EPHB2_M-	EPHB2_M+^b^	P value
	n (%)	n (%)		n (%)	n (%)	
**Lymph nodes**						
-	10 (42)	14 (58)		18 (75)	6 (25)	
+	71 (40)	105 (60)	0.90	130 (74)	46 (26)	0.91
**NHG^c^**						
I	24 (57)	18 (43)		30 (71)	12 (29)	
II	43 (41)	62 (59)		77 (73)	28 (27)	
III	11 (23)	37 (77)	**0.0008**	39 (81)	9 (19)	0.27
**Tumor Size**						
<20 mm	34 (44)	43 (56)		58 (75)	19 (25)	
≥20 mm	45 (38)	74 (62)	0.38	88 (74)	31 (26)	0.83
**ERα**^d^						
-	17 (31)	37 (69)		44 (81)	10 (19)	
+	54 (42)	74 (58)	0.18	93 (73)	35 (27)	0.21
**PgR**^e^						
-	22 (34)	43 (66)		50 (77)	15 (23)	
+	35 (42)	49 (58)	0.33	58 (69)	26 (31)	0.29
**HER2 protein**						
-	74 (44)	93 (56)		124 (74)	43 (26)	
+	7 (21)	26 (79)	**0.01**	24 (73)	9 (27)	0.86
**Treatment**						
Chemotherapy	42 (39)	65 (61)		77 (72)	30 (28)	
Radiotherapy	39 (42)	54 (58)	0.70	71 (76)	22 (24)	0.48
**EPHB2_C**						
-				54 (66)	28 (34)	
+				95 (80)	24 (20)	0.03

a Positive cytoplasmic EPHB2 staining defined as staining >0 in the cytoplasm,

b Positive membranous EPHB2 staining defined as membrane intensity =2,

c Nottingham Grade,

d Estrogen receptor alpha (ERα) cutoff value of 0.05 fmol/μg/DNA,

e Progesterone Receptor.

In bold are the significant P values from the Spearman Rank R test.

Significance was set at the level of P≤0.01 to adjust for multiple comparisons.

#### Survival analysis

Positive cytoplasmic EPHB2 expression was associated with poor patient survival while positive membranous EPHB2 was a good prognostic factor in the univariate analysis. High cytoplasmic EPHB2 expression predicted shorter distant metastasis-free survival, H.R. (95%CI)=1.37 (0.92-2.04), P=0.12 and poor breast cancer survival, H.R. (95%CI)=1.52 (0.10-2.33), P=0.05 (Fig. 4E, 4F). While, high membranous EPHB2 indicated longer distant recurrence-free survival, H.R. (95%CI)=0.66 (0.41-1.05), P=0.08 and longer breast cancer survival, H.R. (95%CI)=0.47 (0.28-0.80), P=0.006 (Fig. 4G, 4H).

Multivariate Cox proportional hazard regression; adjusted for the well-known clinical variables ER, HER2, lymph nodes, tumor size and treatment; showed that membranous EPHB2 was an independent predictor of breast cancer-free survival in addition to lymph nodal status and tumor size. EPHB2 also predicted lower risk to develop metastasis with borderline significance (Table 5). Cytoplasmic EPHB2, albeit indicating higher risk for breast cancer death and metastasis, was not an independent prognostic factor in multivariate analysis.

**Table 5:** Multivariate Cox proportional hazard regression to determine EPHB2 prognostic value in cohort 2

Variables	Breast cancer death		Distant recurrences	
	H.R. (95% CI)^a^	P value	H.R. (95% CI)	P value
**EPHB2_C**				
-	1.0		1.0	
+	1.14 (0.71-1.83)	0.58	1.17 (0.75-1.85)	0.49
**EPHB2_M**				
-	1.0		1.0	
+	0.45 (0.25-0.83)	**0.01**	0.66 (0.39-1.11)	0.12
**ERα**				
-	1.0		1.0	
+	0.69 (0.43-1.11)	0.13	0.82 (0.51-1.31)	0.40
**HER2**				
-	1.0		1.0	
+	1.42 (0.81-2.49)	0.22	1.29 (0.75-2.23)	0.36
**Lymph nodes**				
-	1.0		1.0	
+	2.88 (1.23-6.74)	**0.01**	2.43 (1.1-5.38)	0.03
**Tumor size (mm)**				
<20	1.0		1.0	
≥20	2.12 (1.30-3.47)	**0.003**	1.78 (1.12-2.82)	**0.01**
**Treatment**				
Chemotherapy	1.0		1.0	
Radiotherapy	1.04 (0.67-1.61)	0.87	1.21 (0.79-1.84)	0.38

a Hazard Ratio (H.R.) and confident intervals calculated with the Cox proportional hazard regression model. Significant P≤0.01 in bold.

**Figure 7:**
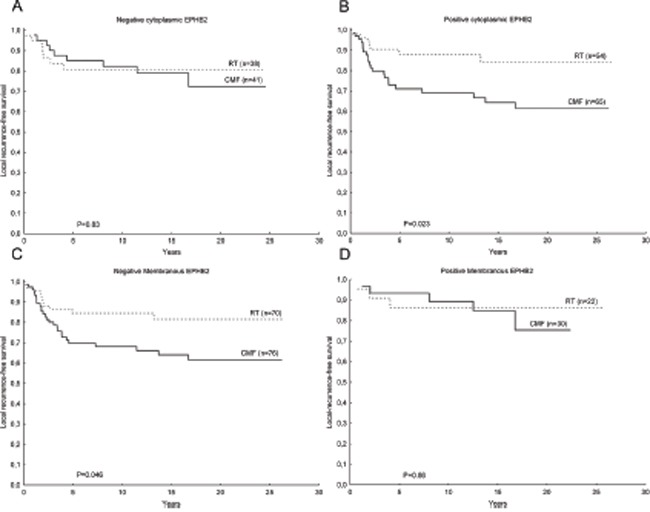
Kaplan-Meier plots showing EPHB2 predictive value in breast cancer Patients with tumors negative for cytoplasmic EPHB2 staining did not benefited from RT compared to CMF in terms of locoregional recurrence-free survival **A.** while the group of patients with positive cytoplasmic EPHB2 staining exhibited a longer locoregional recurrence-free survival time upon RT compared to CMF **B.** (interaction test: P=0.17). The opposite trend was found for EPHB2 membranous staining **C, D.** although the interaction test did not reach statistical significance (P=0.45).

EPHB2 did not have predictive value for patients randomized between radiation treatment (RT) and CMF. Although, patients with positive membranous staining in the tumor cells or negative cytoplasmic staining, did not received a clear benefit from RT compared to CMF in terms of local recurrences-free survival (Fig. 7).

## DISCUSSION

In this study we distinguished a subgroup of patients more likely to relapse with local and distant metastasis and to have a shorter breast cancer free-survival time based on cluster analysis of the *EPH/EFN* mRNA expression levels. *EPHA2*, *EPHA4*, *EFNB1*, *EFNB2*, *EPHB2*, and *EPHB6* were the genes most strongly correlated with the cluster groups. Especially the EPHB2 receptor was the most significantly coupled to patient survival in a multivariate analysis. This result was confirmed in public datasets. Moreover, the EPHB2 protein analysis showed that, in addition to its membranous location, EPHB2 could be found in the cytoplasmic and nuclear cell compartments. Interestingly, membranous and cytoplasmic EPHB2 were negatively correlated indicating opposite patient prognosis: cytoplasmic EPHB2 accounted for shorter breast cancer survival and membranous EPHB2 for good prognosis. Taken together, our results suggest that *EPHB2* mRNA level has clinical value, EPHB2 protein location is important and EPHB2 could be regarded in the context of *EPHA2*, *EPHA4*, *EFNB1*, *EFNB2*, and *EPHB6*.

Hitherto several groups have studied the connections between the EPH family and breast cancer where EPHA2 and EPHB4 are two of the most investigated EPH receptors with potential clinical relevance (reviewed in [4, 17]). A recent study revealed that other family members such as EPHA4, EPHA7 and EPHB6 were negatively correlated with overall patient survival [16]. In our study, based on cluster analysis of mRNA expression levels of *EPHA2, EFNB1, EFNB2, EPHB2, EPHB1, EPHA4, EPHB6, EPHA1, EFNA4, EFNA1, EFNA3, EPHA7, EFNB3, EPHA3, EPHB4, EPHB3 and EFNA5*, we identified two patient clusters: the patients within cluster 2 were characterized by high EPH expression and were more prone to relapse with local metastasis, distant metastasis and had a shorter breast cancer-free survival time. Especially, expression of *EPHA2*, *EPHB2*, *EPHB6*, *EFNB1 and EFNB2* was strongly associated with the cluster variable and coupled to poor outcome. As mentioned before, EPHA2 has been coupled to poor patient survival [15, 18, 19] and to trastuzumab [15] and tamoxifen resistance [20]. Other receptors like *EPHA7* failed to show any clinical value in this study in spite of its previous coupling to shorter recurrence and overall-free survival [16]. *EPHA7* could be affected by promoter methylation, which explains its down regulation in human tumors [21]. Here, we also found low levels of *EPHA7* expression, although detectable in more than 90% of the samples. In general we could detect most of the EPH family members except for *EPHA5*, *EPHA6* and *EPHA8* that were only present in less than 40% of the tumors. *EPHA5* promoter methylation has also been reported. Low EPHA5 is associated with high tumor grade, lymph node metastasis and PgR negative status in breast cancer, indicating that down regulation of EPHA5 could be an important step in tumor progression [22]. Concerning the lower expression of *EPHA6* and *EPHA8*, further studies are needed to unravel their significance. In our study, the lower mRNA levels of *EPHA5*, *EPHA6* and *EPHA8* were not further investigated, as the Ward's algorithm used in the cluster analysis did not take into account genes with more than 50% missing data.

Multivariate analysis revealed that the cluster variable was an independent prognostic marker using the following covariates: treatment, tumor size, HER2 status and ERα. However, adding the *EPHB2* to the multivariate analysis proved that the *EPHB2* was the strongest prognostic factor for most of the survival endpoints. *EPHB2* was one of the genes significantly coupled with the cluster variable and therefore chosen for confirmation in other patient cohorts.

The clinical role of EPHB2 in breast cancer is not well established. High EPHB2 protein expression has been associated with shorter overall survival [23]. The authors reported high EPHB2 cytoplasmic protein and mRNA expression in 51% of the tumors while we found high EPHB2 gene expression in 52% of the cases (cohort 1). Recently EPHB2 was found to be a target of TGFβ3-mediated invasion and migration [24] which is in line with increased EPHB2 protein levels in invasive carcinomas. Also a recent model suggests that EPHB2 could mediate invasion in cells with defective apoptotic machinery via the pro-survival role of autophagy [25]. The EPHB2 invasive properties were kinase-dependent suggesting interactions with an ephrin ligand or another receptor. Indeed, promiscuous interactions between EPH receptors and their ligands with opposite outcomes regarding tumor progression have been reported [11, 26]. For instance a recent study proposed that EPHB6 could decide the fate of the tumor by interacting with other receptors such as EPHB2 and EPHA2 [27]. EPHB6 is a kinase-dead receptor, which may sequester kinase functioning EPH's turning off the oncogenic signaling. Although we found that *EPHB2* was the most promising candidate in our breast cancer cohorts, *EPHB2* together with *EPHB6, EPHA2, EPHA4* and the ligands *EFNB1* and *EFNB2* were important to define prognostic relevant clusters. This information allows speculating that *EPHB2* should be studied in combination with these other factors. Especially the *EPHB6*, which seems to be coupled to invasion upon re-expression in breast cancer cell lines [28, 29] and to adverse prognosis in breast cancer [16] although the *EPHB6* gene seems to be methylated in cancer. To our knowledge, the data regarding the clinical value of *EFNB1 and EFNB2 is scarce* although high ephrin-B1 protein expression seems be involved in the development of brain metastasis from the primary breast tumor [30] and shorter patient overall survival [31].

Furthermore, the prognostic value of *EPHB2* mRNA levels could be statistically demonstrated in the Van de Vijver [32], Uppsala [33] and Karolinska [34] datasets while a trend was seen in the Esserman Perou cohort [35]. We also assessed EPHB2 protein expression in a larger and randomized patient material (cohort 2). EPHB2 was mainly located in the cytoplasm (60% of the tumors) and the cell membrane (26%) although 7% of the tumors presented nuclear staining. Cytoplasmic expression was inversely associated with membranous expression and positively correlated with HER2 protein expression in agreement with the results from cohort 1. Cytoplasmic EPHB2 was also positively correlated with high Nottingham Grade. Cytoplasmic EPHB2 predicted shorter breast cancer survival and tended to indicate shorter metastasis-free survival in univariate analysis. However, membranous EPHB2 was not associated with any known clinical variable and resulted a good prognostic indicator for breast cancer survival in both univariate and multivariate analysis and metastasis-free survival in univariate analysis. These findings suggest that it might be important to make a distinction between cytoplasmic and membranous EPHB2 previous to taking clinical decisions.

Regarding the nuclear localization, EPHB2 has not been reported before as a nuclear protein. However, according to the online tool NLStradamus with default prediction cutoff of 0.5, the EPHB2 has a NLS between aa 1017-1033 corresponding to the aa sequence GKKKGMGKKKTDPGRGR. Otherwise, EPHB4 has been detected in the nucleus of prostate cancer cells [36] and other authors assure that presence of receptor tyrosine kinases in this cell compartment is possible [37] through several mechanisms including receptor internalization upon ligand binding and enzymatic cleavage. According to the free-prediction algorithm PsortII [38], EPHB2 could be present in the Golgi apparatus, endoplasmic reticulum and cell membrane in line with our results. Specificity of the EPHB2 antibody might be an issue. However, the antibody used in this study was extensively validated using several techniques. Still it could be important to consider that EPHB2 have several transcript variants and could undergo posttranslational modifications affecting both protein function and cellular localization. These factors should not be underestimated.

Finally we also took advantage of the randomized study and tested the EPHB2 predictive value finding an inverse trend between membranous EPHB2 expression and response to radiotherapy.

In summary we found that the EPH receptors and the ephrin ligands are potential clinical candidates. Especially *EPHA2*, *EPHA4*, *EFNB1*, *EFNB2*, *EPHB2*, and *EPHB6* and their co-expression in breast cancer. EPHB2, although poorly investigated, has shown to be a promising prognostic marker in breast cancer but more studies on its protein expression and localization are still encouraged.

## MATERIALS AND METHODS

### Clinical materials

All tumor samples were collected during the Stockholm clinical trial (1976-1990) [39]. The trial included premenopausal and postmenopausal women with a unilateral, operable breast cancer. The surgery procedure was modified radical mastectomy. Further inclusion criteria were either histologically verified lymph node metastasis or a tumor diameter, exceeding 30 mm, measured on the surgical specimen. Patients received either adjuvant chemotherapy or radiotherapy, and both groups were randomized to tamoxifen or no endocrine treatment. Tamoxifen was administered postoperatively at a dose of 40 mg daily for 2 or 5 years. Patients in the chemotherapy group received 12 courses of cyclophosphamide, methotrexate, 5-fluorouracil (CMF) according to the original Milan protocol (100 mg/m^2^ cyclophosphamide orally at days 1-14, 40 mg/m^2^ methotrexate and 600 mg/m^2^ 5-fluorouracil intravenously on days 1 and 8). However, in the first 18 months of the trial, 10-15 mg chlorambucil was administered orally on days 1-8 instead of cyclophosphamide and to avoid dose reductions up to 18 months treatment time was allowed for the 12 courses. Patients randomized to radiation treatment (RT), received a dose of 46 Gy with 2 Gy per fraction 5 days a week. Total treatment time was about 4.5 weeks and the target volume included the chest wall, the axilla, the supraclavicular fossa and the internal mammary nodes. In this study we included two patient cohorts from the Stockholm trial (Fig. 1): Cohort 1: originally comprised 679 postmenopausal patients. From those tumor tissue was available from 282 and RNA from 90 patients. In this study we included 70 patients with good RNA quality and from these, 65 patients with lymph nodal infiltration were analyzed. Some clinical variables used here were described in previous studies: ER [39], ERBB2 gene amplification [40], S-phase fraction, HER2 protein levels [41], pAKT [41]. Cohort 2 was initially composed of 547 premenopausal patients and from these, 216 patients, with available tumors, were included. These tumors were paraffin embedded and available on TMA allowing detection of protein expression by immunohistochemistry. The characteristics of the patients included in cohorts 1 and 2 did not significantly differ from all patients included in the Stockholm trial (Supplementary Table S1). The retrospective studies on tumor tissues have been approved by the Research Ethics Committee at the Karolinska Institute (dnr 97–451), with amendments.

### Cell lines

Breast cancer cell lines: MDA-MB-231 (HTB-26), MDA-MB-468 (HTB-132) and T47-D (HTB-133) and colorectal cancer cells: HCT116 (CCL-247) and SW620 (CCL-227) were purchased from the American Type Culture Collection (ATCC) and tested for mycoplasma using the PCR Mycoplasma Test Kit I/C from PromoKine (PromoCell GmbH, Germany). Breast cancer cells were cultured in Dulbecco's Modified Eagle's Medium supplemented with 4% fetal bovine serum and penicillin and streptomycin. SW620 cells were cultured in Eagle's Minimum Essential Medium supplemented with 2 mM L-glutamine, 10% Fetal Bovine Serum (FBS) and HCT116 cells in Mc Coy's medium supplemented with 10% FBS. The mouse brain cell lysate was a kind gift from Ravi Kumar Dutta.

## ANTIBODY VALIDATION

### siRNA and immunoblot

The HCT116 and SW620 cells were transfected with a pool of EPHB2 siRNAs or a negative control siRNA in the Amaxa Nucleofector 2B and the Nucleofection Mix Solution V (Lonza) following manufacturer's instructions. Transfected cells were harvested after 7 days. siEPHB2 Silencer Select s4740 + s4741 were pooled at 300nM (ThermoFisher Scientific). The AllStar Negative Control (SI03650318, Qiagen) was also used at 300nM. Upon transfection, cell lysates were prepared in RIPA buffer containing proteases inhibitors (Complete Mini, Roche) and the protein concentration was measured with the Bicinchoninic Acid Assay (Pierce Biotechnology). Total proteins (30 μg/well) were loaded in the gel. For the immunoblot, primary antibodies, rabbit anti-EPHB2, 1:500 (Cat # AP7623d, Nordic BioSite) or anti-beta actin, 1:1000 (Cell Signaling) were diluted in blocking buffer (TBS-0.1% Tween20/5% milk) and incubated at 4°C overnight. The secondary antibodies (DAKO) conjugated with horseradish peroxidase (HRP) were incubated for 1 h at room temperature. Proteins were visualized with HyGLO chemiluminescent HRP-antibody detection reagent and developed with BioMax light film (Carestream Health).

### Paraffin embedding

HCT116 cells at 80-90% confluence were harvested and the pellet fixed with 4% formaldehyde at room temperature for 25 min. The cells were stained with hematoxylin and centrifuged at 1200 rpm for 2 min followed by progressively dehydration in ethanol at 70% (overnight), 95% (1 h) and 99.5% (1 h). Finally, xylene was added to the pellet for 30 min and after centrifugation the cell pellet was paraffin embedded at 56°C overnight. The embedded cells were cut in 4 μm slices, using a microtome and the slides stained following the immunohistochemistry protocol described below.

### Gene expression profile

Quantitative real-time RTPCR was performed using aTaqman^®^ Array Micro Fluidics Cards (Applied Biosystems, Life technologies, UK) that included 21 EPH family members: *EPHA1-A8, EPHB1-B4* and *EPHB6, EFNA1-A5, EFNB1-B3*; and two endogenous controls: glyceraldehyde-3-phosphate dehydrogenase (*GAPDH*) and *hypoxanthine phosphoribosyltransferase 1* (*HPRT1*) (details of the array are compiled in Supplementary Table S2). Each card included 8 samples: 7 breast tumors and an internal standard consisting of a pool of 7 cell lines. Samples were run in duplicates.

A cDNA equivalent of 200 ng RNA was adjusted to 52 μL with RNase free water and mixed with 51μL of TaqMan^®^ Universal Master Mix II with uracil-DNA glycosylase (UNG) (Applied Biosystems, Life technologies, UK). The mixture was loaded into one slot of a TaqMan^®^ Array Micro Fluidics Card. The PCR reaction was run in a 7900HT Fast time PCR system (Applied Biosystems, Life technologies, UK).

Relative mRNA expression levels of target genes within a sample was calculated with the ΔΔ*C*_T_ method [42] using RQ manager version 1.2 (Applied Biosystems, Life technologies, UK). The cell line pool was used as reference sample, and the *HPRT1* gene was chosen as endogenous control due to its low expression variation as confirmed with the geNorm algorithm embedded in the StatMiner version 4.2 software (Integromics, Spain). Non-amplified wells and duplicates with SD>0.5 were omitted in the RQ manager.

### Immunohistochemistry (cohort 2)

Tissue microarray (TMA) slides including 216 breast cancer patient samples from the Stockholm trial were incubated for 2 hours at 60°C prior to de-paraffinization and antigen retrieval in a PT-Link system (DAKO, Denmark). Antigen retrieval was performed at pH 6.0 for 20 min at 97°C. A washing buffer, consisting in TBS-0.1% BSA, was used previous to inactivation of endogenous peroxidase in 3% H_2_O_2_ for 10 min. Unspecific binding was blocked with serum-free protein block (Background Sniper, Biocare Medical) for 10 min in a moisture chamber. The rabbit anti-EPHB2 antibody (1:300) was incubated overnight at 4°C. The HRP conjugated-secondary antibody (Envision+System-HRP Labelled-Polymer anti Rabbit, DAKO, Ref#4002) was incubated for 30 min and the chromogenic agent and substrate was a DAB/H_2_O_2_ solution. Cell nuclei were counterstained with Mayer's Hematoxylin prior to stepwise dehydration with ethanol, 40%, 70%, 95%, 99.5% and tissue clear. The TMA slides were mounted with Pertex and images were acquired with an Aperio Scanscope AT Turbo (Leica Biosystems) with 20x/0.75 NA Plan Apo and with 20X magnification. The software Aperio ImageScope v.12 was used for image analysis.

### IHC scoring

Staining was evaluated on three separate core biopsies by two individual observers blinded to the clinical data. The sections were re-evaluated upon disagreement. EPHB2 was mainly visualized in the cell membrane and the cytoplasm. Few tumors also presented nuclear staining. The cytoplasmic (C) and membrane (M) staining were based on intensity (negative =0, weak=1 and strong=2). The cut off for positive cytoplasmic staining was C>0 and for positive membranous staining, M=2.

### Statistical analyses

The statistical analyses of relative mRNA expression levels in cohort 1 were performed in R version 3.0.2 (R Core Team (2013). R: A language and environment for statistical computing. R Foundation for Statistical Computing, Vienna, Austria. URL http://www.R-project.org/). Only patients with lymph node infiltration were included in the statistical analyses, and previous to the analysis the data was cleaned by only including the genes with detected expression in more than 60% of the tumor samples (42/70).

Hiearchical clustering of relative mRNA expression levels was performed on scaled data with mean value = 0 and standard deviation = 1 using the Complete linkage method and Eucledian distance. Cox Proportional Hazard regression was used in univariate and multivariate analyses to test if relative mRNA expression levels correlated with the endpoints breast cancer-survival (period from surgery until death due to breast cancer is reported), local recurrence-free survival (time from surgery until local recurrence is detected) and metastasis-free survival (time from surgery until distant metastasis is detected). Patient survival was represented with the Kaplan-Meier plots.

The statistical analysis of EPHB2 protein expression in cohort 2 was performed with Statistica 64 version 12.0 software (StatSoft. Inc, USA). Relationship with known clinical variables in breast cancer was tested with the Spearman Rank correlation test. Cox regression was used in univariate and multivariate analyses to test if there was an independent association between EPHB2 protein expression and the presence of distant metastases, local metastasis or death due to breast cancer. The survival analysis to estimate probabilities for metastasis-free survival (time from surgery until distant metastasis is detected), local recurrence-free survival (time from surgery until local recurrence is detected) and breast cancer-free survival (period from surgery until death due to breast cancer is reported) were calculated by comparing survival in multiple samples and represented with the Kaplan-Meier plots. When needed significance was set to p-value P<0.01 to compensate for multiple comparisons.

### Public gene expression datasets

The EPHB2 results were validated in the following gene expression datasets: van de Vijver (n=295) [32], Uppsala (GSE3494, n=236) [33], Karolinska Institute (KI) (GSE1456, n=159) [34] and Esserman, Perou (GSE22226, n=147) [35]. For the statistical analysis gene expression data were divided into quartiles (q) where q1-3 was defined as low expression and q4 was high expression (Van de Vijver and Uppsala) or q1 was low vs. q 2-4 high (Esserman-Perou and KI). When several probes were used to detect *EPHB2* mRNA expression (KI and Uppsala) and the probes were positively correlated, the average of the gene expression data was used for the analysis.

## SUPPLEMENTARY DATA FIGURE AND TABLES


