# Supplementary material for: EPH/ephrin profile and EPHB2 expression predicts patient survival in breast cancer
Source: Oncotarget. 2016 Feb 8;7(16):21362–80. doi: 10.18632/oncotarget.7246 (PMC5008291; doi:10.18632/oncotarget.7246)
Supplement: Supplementary file 1 [file oncotarget-07-21362-s001.pdf]

SUPPLEMENTARY DATA

Protein identification by in-gel digestion, peptide analyses by LC-MS/MS and data analysis

In –gel digestion was performed generally as described by Shevchenko [43]. *Briefly:* the Coomasie-blue stained protein bands corresponding to *EPHB2* were excised, distained, reduced by 10mM DTT, alkylated by 55 mM iodoacetamide in the dark*ness* and digested by 0.005 µg/µL trypsin (Thermo scientific, Rockford, IL, USA) over night at 37°C. Peptides were dried, dissolved in 0.1% formic acid and analyzed by liquid chromatography-mass spectrometry (LC-MS/MS). Peptides were separated by reverse phase chromatography on C18 columns with 5 µm particle size (20 mm×100 µm pre column followed by a 100 mm×75 µm column) (NanoSeparations, Netherlands) at a flow rate 300 nL/min in a 45 min linear gradient going from 0.1% formic acid/water to 0.1% formic acid/100% acetonitrile. Data acquisition was performed using a LTQ Orbitrap Velos Pro hybrid mass spectrometer (Thermo Fisher Scientific, San Jose, CS, USA). Data files were analyzed by the Proteome Discoverer 1.4 (Thermo Scientific) and the search algorithm SEQUEST HT was used against UniProt Homo sapiens database. Trypsin was used as the enzyme with one-missed cleavages being allowed and carbamidomethylation of cysteine residues was set as static modification. The precursor mass tolerance was set to 10 ppm with 0.6 Da fragment mass tolerance. The result was filtered with peptide confidence value “high” and target false discovery rate 0.01.

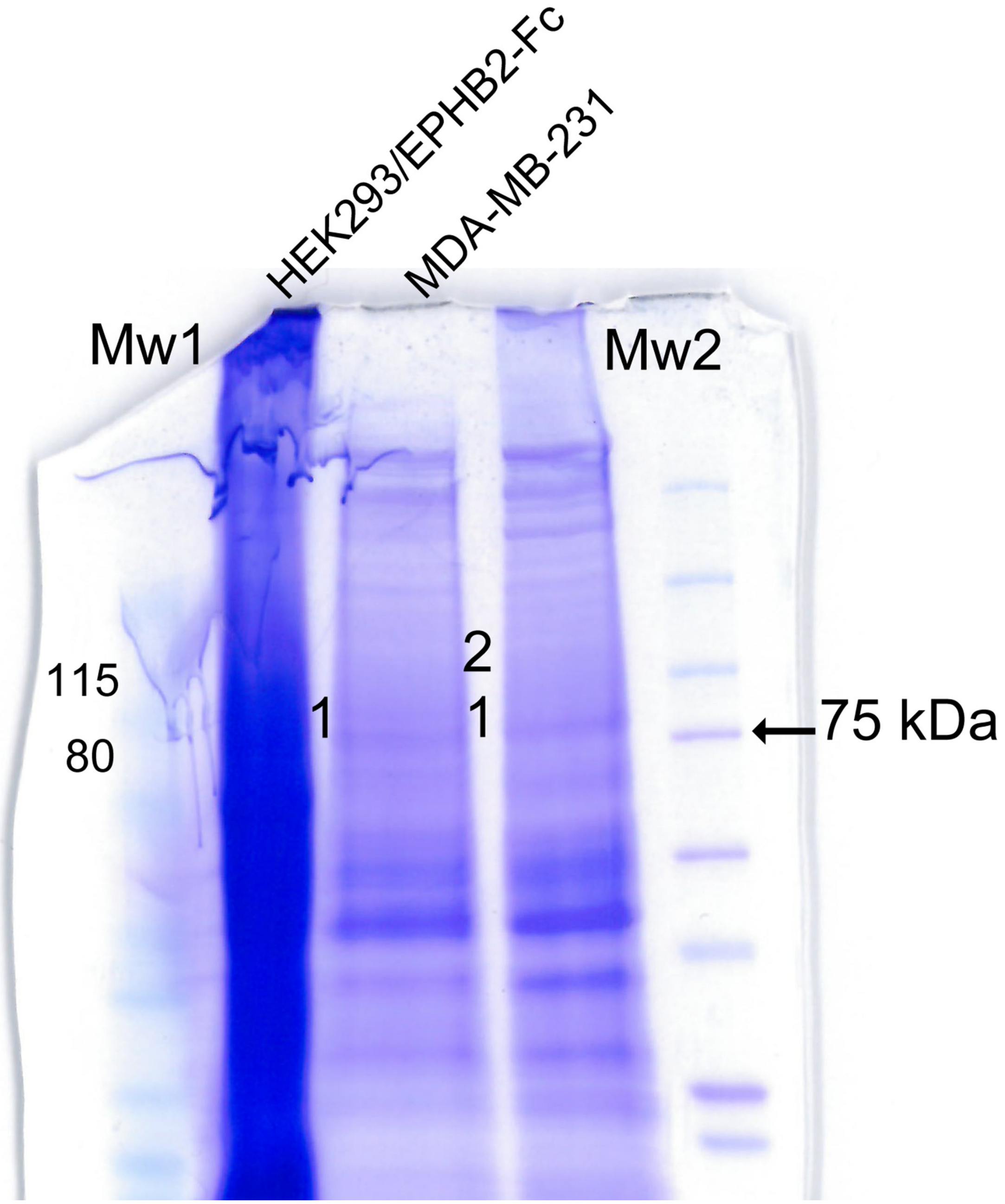

Supplementary Figure S1: Coomasie Blue staining of total proteins from MDA-MB-231 and HEK293/EPHB2-Fc (positive control) cells. Numbers 1 and 2 correspond to the excised bands used for in-gel digestion. The right arrow indicates the 75kDa band 1 according to the precision plus protein standards (Mw2). The Benchmark protein ladder was also used (Mw1).

Supplementary Table S1: Comparing the patient characteristics between the Stockholm trial and the two cohorts included in this study

Patient	Cohort 1 (postmenopausal)					Cohort 2 (premenopausal)				
characteristics	n=679		n=70			n=547		n=216		
	n	%	n	%	Chi^2	n	%	n	%	Chi^2
**Age**										
<50	19	3	4	6		465	85	188	87	
50-59	344	51	43	61		82	15	28	13	P=0.68
60-69	306	45	23	33						
≥70	9	1	0	0	0.19					
**Lymph nodes**										
Negative	79	12	5	7		66	12	28	13	
Positive	538	79	65	93	P=0.15	481	88	188	87	P=0.83
Missing	62	9								
**Tumor Size (mm)**										
≤20	295	43	26	37		215	39	81	37	
>20	350	52	44	63	P=0.24	316	58	129	60	P=0.77
Missing	34	5				16	3	6	3	
**ERα**										
Negative	139	23	17	24		144	26	58	27	
Positive	456	74	53	76	P=0.96	341	62	137	63	P=0.94
Missing	84	3				62	12	21	10	
**Treatment**										
Radiotherapy	308	55	32	46		256	47	99	46	
Chemotherapy	371	45	38	54	P=0.20	291	53	117	54	P=0.89
**tamoxifen**										
No	330	49	29	41						
Yes	349	51	41	59	P=0.26					

Supplementary Table S2: TaqMan® Array probes included in the *EPH/EFN* array

Gene Symbol	*Assay ID*
EFNA1	*Hs00358886_m1*
EFNA2	*Hs01023290_m1*
EFNA3	*Hs00191913_m1*
EFNA4	*Hs00193299_m1*
EFNA5	*Hs00157342_m1*
EFNB1	*Hs00270004_m1*
EFNB2	*Hs00187950_m1*
EFNB3	*Hs00154861_m1*
EPHA1	*Hs00178313_m1*
EPHA2	*Hs00171656_m1*
EPHA3	*Hs00739096_m1*
EPHA4	*Hs00177874_m1*
EPHA5	*Hs00300724_m1*
EPHA6	*Hs00297133_m1*
EPHA7	*Hs00177891_m1*
EPHA8	*Hs00184126_m1*
EPHB1	*Hs01057849_m1*
EPHB2	*Hs00362096_m1*
EPHB3	*Hs00177903_m1*
EPHB4	*Hs00174752_m1*
EPHB6	*Hs01071144_m1*
GAPDH	*Hs99999905_m1*
HPRT1	*Hs02800695_m1*

Supplementary Table S3: EPHB2 tryptic peptides identified by LC-MS/MS from MDA-MB-231 and HEK293/EPHB2-Fc band 1

Peptide Sequenced	XCorr	m/z, amu	Charge state	EPHB2 isoforms matching		
				Protein name	Accession number	Uniprot Theoretical Mw (kDa)
VDTIAADESFSQVDLGGR	5.1	940.45	2	Ephrin type-B receptor 2<br>Isoform 2 of Ephrin type-B receptor 2<br>Isoform 3 of Ephrin type-B receptor 2<br>EPH receptor B2<br>EPHB2 protein	P29323<br>P29323-2<br>P29323-3<br>B1AKC9<br>Q6NVW1	117.4<br>109.8<br>110.0<br>105.5<br>53.2
ETFNLYYYEADFDSATK	3.7	1038.96	2			
